# Piezo1 Participated in Decreased L-Type Calcium Current Induced by High Hydrostatic Pressure *via*. CaM/Src/Pitx2 Activation in Atrial Myocytes

**DOI:** 10.3389/fcvm.2022.842885

**Published:** 2022-02-17

**Authors:** Yuan Fang, Qian Li, Xin Li, Guan-Hao Luo, Su-Juan Kuang, Xue-Shan Luo, Qiao-Qiao Li, Hui Yang, Yang Liu, Chun-Yu Deng, Yu-Mei Xue, Shu-Lin Wu, Fang Rao

**Affiliations:** ^1^Guangdong Cardiovascular Institute, Guangdong Provincial People's Hospital, Guangdong Academy of Medical Sciences, Guangzhou, China; ^2^Guangdong Provincial Key Laboratory of Clinical Pharmacology, Research Center of Medical Sciences, Guangdong Provincial People's Hospital, Guangdong Academy of Medical Sciences, Guangzhou, China

**Keywords:** atrial fibrillation, L-type calcium channel, high hydrostatic pressure (HHP), Piezo1, calmodulin, Src kinase

## Abstract

Hypertension is a major cardiovascular risk factor for atrial fibrillation (AF) worldwide. However, the role of mechanical stress caused by hypertension on downregulating the L-type calcium current (I_Ca,L_), which is vital for AF occurrence, remains unclear. Therefore, the aim of the present study was to investigate the role of Piezo1, a mechanically activated ion channel, in the decrease of I_Ca,L_ in response to high hydrostatic pressure (HHP, one of the principal mechanical stresses) at 40 mmHg, and to elucidate the underlying pathways. Experiments were conducted using left atrial appendages from patients with AF, spontaneously hypertensive rats (SHRs) treated with valsartan (Val) at 30 mg/kg/day and atrium-derived HL-1 cells exposed to HHP. The protein expression levels of Piezo1, Calmodulin (CaM), and Src increased, while that of the L-type calcium channel a1c subunit protein (Cav1.2) decreased in the left atrial tissue of AF patients and SHRs. SHRs were more vulnerable to AF, with decreased I_Ca,L_ and shortened action potential duration, which were ameliorated by Val treatment. Validation of these results in HL-1 cells in the context of HHP also demonstrated that Piezo1 is required for the decrease of I_Ca,L_ by regulating Ca^2+^ transient and activating CaM/Src pathway to increase the expression of paired like homeodomain-2 (Pitx2) in atrial myocytes. Together, these data demonstrate that HHP stimulation increases AF susceptibility through Piezo1 activation, which is required for the decrease of I_Ca,L_ *via*. the CaM/Src/Pitx2 pathway in atrial myocytes.

## Introduction

Atrial fibrillation (AF), one of the most frequent cardiac arrhythmias, is associated with increased risks of stroke and heart failure, and thus, continues as a burden to healthcare systems worldwide (1). Currently available therapies include antiarrhythmic drugs and catheter ablation. However, there are many limitations that adverse effect and limited efficacy for the former and potential complications for the latter (2). Hence, a better understanding of the mechanisms underlying substrate formation may provide promising and novel insights into the treatment of AF. Hypertension is a common risk factor for AF. As a modifiable risk factor, management of high blood pressure (BP) can reduce the risks of new-onset AF and recurrence after cardioversion or ablation (3, 4). Previous studies have revealed that mechanical stress can lead to electrical remodeling of AF (5–8), which is characterized by a decrease in L-type calcium current (I_Ca,L_) and shortening of the action potential duration (APD) (9, 10). However, the specific molecular mechanisms underlying the perception and translation of mechanical stress into a cellular response in atrial myocytes remains unclear.

Mechanosensitive ion channels (MSCs) participate in mechanotransduction, an ancient sensing mechanism, responsible for the conversion of mechanical stimuli into biochemical responses (11). Piezo proteins, a recently discovered family of excitatory ion channels directly gated by mechanical forces, are involved in various mechanotransduction processes, such as mechanosensory pain and touch (12, 13). In the cardiovascular system, Piezo1 is required for angiogenesis, vascular maturation, and the baroreceptor reflex (14–18), Piezo1 required for the release of nitric oxide for blood pressure control mediates fluid shear stress sensing in endothelial cells (19), and is activated by stretch, involved in hypertension-dependent arterial remodeling in smooth muscle cells (20). It is also found to increase in ventricular myocytes under acute myocardial infarction and can be inhibited by ARB therapy (21). However, the ability of Piezo1 in atrial myocytes to perceive mechanical stress and its role in atrial electrical remodeling of AF induced by hypertension, especially the regulation of I_Ca,L_, remains unclear. A past study has investigated the role of atrial stretch, while ignoring hydrostatic pressure (22), thus the focus of the present study is hypertension-induced changes in mechanical stress.

Piezo1 possesses transmembrane triskelions to integrate exquisite mechanosensitivity with the regulation of Ca^2+^ influx (23). At the same time, a variety of Ca^2+^ binding proteins are used for Ca^2+^ signal transduction, in which Calmodulin (CaM), a ubiquitous Ca^2+^-sensing protein, plays a central role. CaM, which is expressed by all eukaryotic cells, couples Ca^2+^ signaling to multiple effector molecules to mediate appropriate cellular responses (24, 25). However, further studies are needed to determine whether CaM is activated by an influx of Ca^2+^ through Piezo1 and potential involvement in the decrease of I_Ca,L_ in atrial myocytes. Previous studies have confirmed that CaM activates Src, a non-receptor tyrosine kinase, *via*. regulatory sites (26–28). Src functions in multiple cellular processes, and participates in the occurrence of AF. Previous studies have indicated that inhibition of Src can increase I_Ca,L_ in human atrial myocytes, suggesting that I_Ca,L_ can be decreased by Src kinase in atrial myocytes (29, 30). A previous study by our group also suggested that Src was involved in the decrease in I_Ca,L_ in atrial myocytes under conditions of high hydrostatic pressure (HHP) at 40 mmHg (31). However, the mechanism used by HHP to activate Src is unclear.

Therefore, the aim of the present study was to explore the functional role of Piezo1 on the decrease of I_Ca,L_ in atrial myocytes in response to HHP and identify the underlying signaling pathways. The results show that Piezo1, as a functional Ca^2+^-permeable MSC in atrial myocytes activated by HHP, is associated with decreased I_Ca,L_ through the CaM/Src/paired like homeodomain-2 (Pitx2) signaling pathway.

## Methods

### Patients

The study protocol was approved by the Research Ethics Committee of the Guangdong Provincial People's Hospital (Guangzhou, Guangdong Province, China; Guangdong Academy of Medical Sciences approval no. GDREC2017111H) and conducted in accordance with the ethical principles regarding Medical Research Involving Human Subjects described in the Declaration of Helsinki. All patients provided signed informed consent. Patients with any infectious disease were excluded from the study.

Left atrial appendages (LAAs) were acquired from 10 patients with chronic AF (≥6 months) during open-heart surgery conducted in Guangdong General Hospital and 10 patients with normal sinus rhythm (SR) as a control, cut into pieces, and stored at −80°C until analyzed. The 10 chronic AF patients and 10 with normal SR were matched by sex distribution, age, type of valve disease, and medication status.

### Animals

The animal study protocol was approved by the Research Ethics Committee of Sun Yat-sen University (Guangzhou, China; ethic code: SYSU-IACUC-2020-000220) and conducted in accordance with the National Institutes of Health Guide for the Care and Use of Laboratory Animals (NIH publication no. 85-23, revised in 1996). Male spontaneously hypertensive rats (SHRs) and age-matched Wistar rats (30–32 week old) were obtained from Vital River Laboratory Animal Technology Corp (Beijing, China, production license number: SCXK 20160006). SHRs randomly received either oral administration of the angiotensin type 1 receptor (AT1R) blocker valsartan (SHR + Val, 30 mg/kg/day, *n* = 12) or an equal volume of saline (*n* = 12) for 8 weeks. Wistar rats (*n* = 12) were used as controls. Tail-cuff plethysmography was used to monitor BP before the electrophysiology study. Afterwards, all rats were euthanized with carbon dioxide and hearts were collected for analysis.

### Electrophysiology Analysis

The rats were anesthetized by an intraperitoneal injection of 3% pentobarbital (45 mg/kg) anesthetized rats before all electrophysiological measurements and additional doses were administered when required throughout the experiment. A heating pad (RWD Life Science Co., Ltd., Shenzhen, China) was used to monitor and maintain temperature at 37–38°C.

#### Electrocardiogram (ECG) Recordings

ECG data were processed with the iWorx Data Acquisition and Analysis System (https://iworx.com/). ECG limb leads I and II were monitored continuously using subcutaneous platinum needle electrodes. P-wave duration (PWD) and PR interval were evaluated on the surface ECG as an average of 5 consecutive beats.

#### AF Inducibility

AF was induced by 15 s atrial burst pacing delivered at 20 ms basic cycle lengths, 2-fold diastolic pacing threshold, and 1 ms pulse width. This procedure was repeated 10 times for each class. AF was considered induced by fragmented and rapid atrial electrograms with an irregular ventricular rhythm persisting for more than 1 s after burst pacing. The interval between initiation and spontaneous termination of AF determined the AF duration. The percentage of successful inductions of AF defined AF inducibility.

### Preparation of rat Atrial Myocytes

Atrial myocytes were isolated from LAAs or the left atrial (LA) tissue of rats. In brief, the perfusate (0.65 g HELP-Na, 2.75 g minimum essential medium, 0.225 g NaHCO_3_, 250 ml distilled water, and pH 7.35) and enzymatic hydrolysate (40 ml perfusion solution containing 0.02 g collagenase and 0.04 g bovine serum albumin) were preheated and then used to isolate rat atrial myocytes with a Langendorff constant-flow perfusion device. Cells were dissociated and suspended in a solution containing (in mM): KCl 40, K-glutamate 50, KOH 20, KH_2_PO_4_ 20, Taurine 20, MgCl_2_·6H_2_O 3, glucose·H_2_O 10, EGTA 0.5, and HEPES 10 (pH 7.4 with KOH). The sediment cells were appropriate for experimentation within 8 to 10 h.

### Immunohistochemistry

Rat hearts were formalin-fixed and paraffin-embedded using standard protocols, and cut into 4 μm-thick sections, which then were deparaffinized, rehydrated, washed with phosphate-buffered saline (PBS), mounted on glass slides, and incubated overnight with a rabbit polyclonal antibody (Ab) against Piezo1 (dilution, 1:50). The next day, the slides were washed three times for 5 min with Tris-buffered saline with Tween™ 20 and then incubated with a horseradish peroxidase-labeled goat antirabbit secondary Ab. After washing three times, the sections were incubated with diaminobenzidine tetrahydrochloride in PBS containing H_2_O_2_ for 15 min. Following a final wash with distilled water, the slides were observed under a light microscope.

### Culture of HL-1 Cardiomyocytes

Mouse cardiac muscle HL-1 cells were provided by Dr. William Claycomb (Louisiana State University Health Science Center, New Orleans, LA, USA), cultured in Claycomb medium containing 10% fetal bovine serum, 100 μM noradrenaline, and 2 mM L-glutamine in flasks pre-coated with gelatin and fibronectin (Sigma, St Louis, MO, USA), then incubated at 37 °C under an atmosphere of 5% CO_2_/95% air. Afterwards, the cells were exposed to different hydrostatic pressures (0, 20, and 40 mmHg) for 24 or 48 h using a device developed in-house (patent no. 201420109263.1, China), as previously described (31).

### Whole-Cell Patch Clamp Recording

Experiments were performed 4 h after obtaining rats atrial cells or 2–3 h after digestion of the cells adhering to the plate wall. After perfusion with extracellular solution, the membrane capacitance and I_Ca,L_ of the cells were measured with a whole-cell voltage clamp, while the action potential (AP) of a single cell was measured using a current clamp.

The internal solution for I_Ca,L_ measurements was composed of (in mM) TEA–Cl 20, CsCl 100, Na_2_GTP 0.4, ATP-Na_2_ 5, HEPES 10, and EGTA 10 (pH 7.2 with Tris). The external solution contained (in mM) CsCl 5.4, Choline-Cl 126, MgCl_2_·6H_2_O 1, NaH_2_PO_4_·2H_2_O 0.33, HEPES 10, Glucose·H_2_O 10, and CaCl_2_·2H_2_O 2, pH 7.4 (CsOH). The internal solution for AP measurements contained (in mM) MgCl_2_·6H_2_O 1, KCl 140, EGTA 5, HEPES 10, and Na_2_-ATP 5 (pH 7.2, KOH). The external solution contained (in mM) KCl 5.4, NaCl 136, D-glucose 10, MgCl_2_·6H_2_O 1, CaCl_2_ 1.8, HEPES 10, and NaH_2_PO_4_·2H_2_O 0.33 (pH 7.4 with NaOH).

After applying positive pressure inside the patch pipette, the patch-clamp pipettes were placed in the bath solution. When entering into the bath solution, the tip resistance was 2–5 ΩM and the tip potential was set to 0 before the pipette came in contact with the cell. After gigaseal formation, the whole-cell configuration was established by gentle suction or an electrical shock. Pipette capacitance, series resistance, as well as whole-cell capacitance were compensated before the recording. Current signals were recorded using an EPC10 amplifier (HEKA Elektronik GmbH, Lambrecht, Germany) driven by PatchMaster software (HEKA Elektronik GmbH). Series resistances of 2–20 MΩ was electrically compensated by 70–80% to minimize the voltage drop across the clamped membrane. During the recording, the current was maintained at a constant value. All experiments were conducted at room temperature (25 ± 1°C).

### Western Blot Analysis

After treatment, LA tissues and HL-1 cells were lysed, and the supernatants were collected and centrifuged for measurement of protein concentrations. Samples were adjusted with loading buffer to attain equal protein volumes and heated at 55°C for 10 min (for Piezo1 and Cav1.2) or 100°C for 10 min (for Src and Pitx2) for denaturation. According to standard protocols, the treated protein samples (15–30 μg) were separated by electrophoresis with 10% SDS–polyacrylamide gels and transferred to PVDF membranes, which were blocked with 5% non-fat milk for 1 h at room temperature and then incubated overnight at 4°C with primary rabbit polyclonal Abs against Piezo1, Cav1.2 (dilution, 1:1000; Alomone Labs, Jerusalem, Israel); and Src (1:1000; Abcam, Waltham, MA, USA;) and mouse polyclonal Abs against Pitx2 (1:1000; Cloud-Clone Corp., Wuhan, Hubei, China) and glyceraldehyde 3-phosphate dehydrogenase (GAPDH) or β-actin (1:5000; Cell Signaling Technology, Inc., Beverly, MA, USA). The next day, the membranes were washed three times and then incubated with horseradish peroxidase-conjugated secondary Abs against mouse immunoglobulin (Ig)G (Cell Signaling Technology, Inc.) or rabbit IgG (Abcam) for 1–2 h. Blots were visualized using an electrochemiluminescence detection reagent (Merck Millipore, Darmstadt, Germany). Bands were quantified as the ratio of the target protein to the internal reference (GAPDH or β-actin).

### Ca^2+^ Imaging

HL-1 cells were plated on 0.1% gelatin-coated confocal dishes. For Ca^2+^ flux measurements, the cells were loaded with Fluo-4/AM (3 μM), a Ca^2+^ indicator, for 30 min. After washing, the cells were treated with Tyrode's solution containing CaCl_2_ (2 mM) followed by 10 μM Yoda1 to record the Ca^2+^ influx mediated by Piezo1. The fluorescence was observed with a confocal laser scanning microscope (SP5-FCS; Leica Microsystems GmbH, Wetzlar, Germany) and the fluorescence emission was monitored at a wavelength of 525 nm. The fluorescence intensity was corrected for background fluorescence of cell-free areas. The Δ*F/F* ratio was calculated for further analysis. Here, Δ*F* is the change in stimulation-evoked fluorescence and *F* is baseline fluorescence monitored immediately before stimulation.

### Data and Statistical Analysis

All data were expressed as mean ± SEM. Statistical significance between groups were analyzed using the Fisher's exact test, one-way analysis of variance (ANOVA) and two-tailed Student's *t*-test, where appropriate. *p* < 0.05 indicated statistical significance.

## Results

### Expression of Piezo1 and Pathway-Related Proteins in LAAs of Patients With SR vs. AF

**Figure 1:**
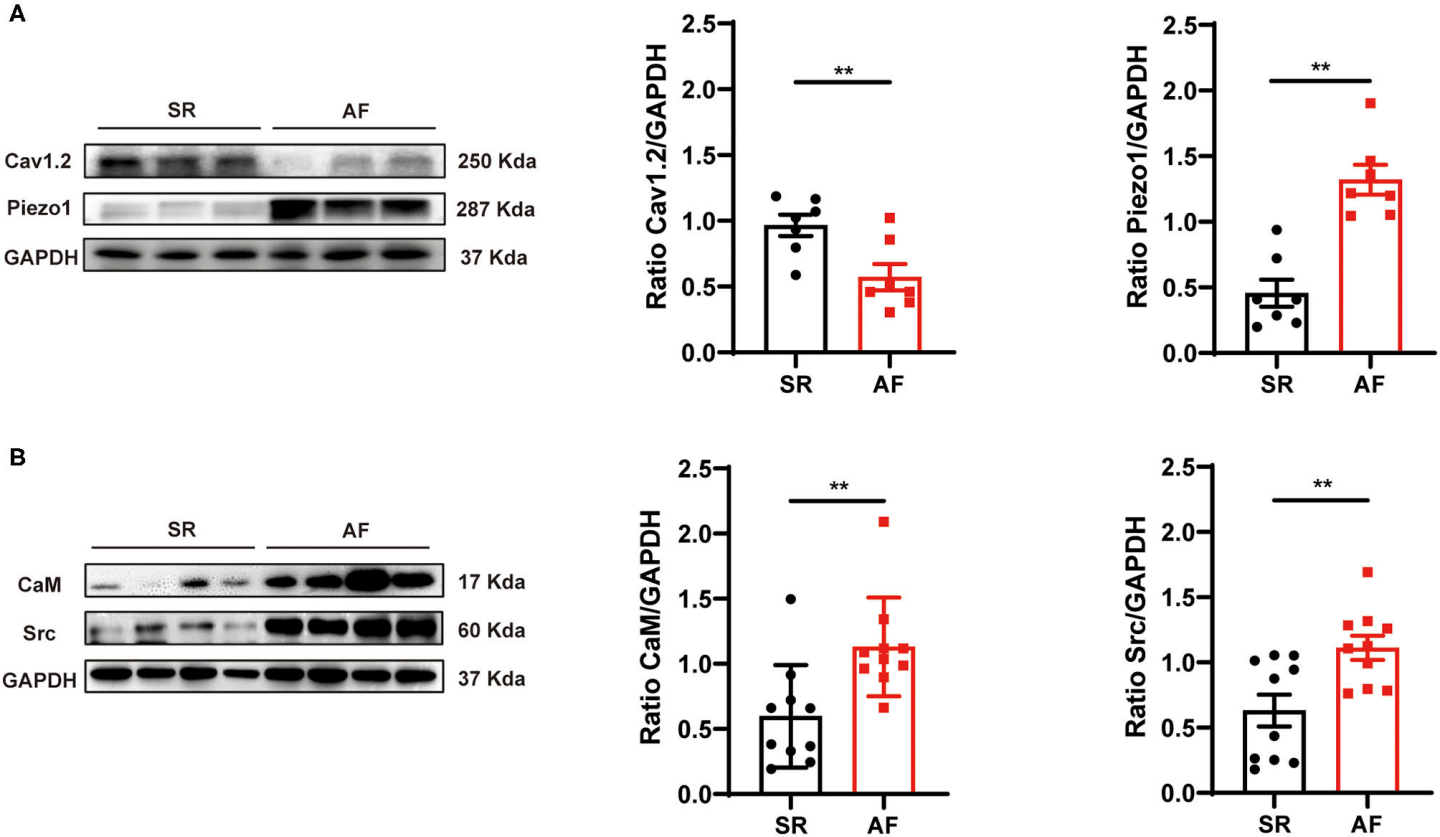
Protein expression levels of Cav1.2, Piezo1, CaM, and Src in human LAA tissues. **(A)** Representative western blots and densitometric analysis of Cav1.2 and Piezo1 proteins in LA tissues of AF patients and those with SR. **(B)** Representative western blots and densitometric analysis of CaM and Src protein in LA tissues of AF patients and those with SR. GAPDH was the internal control. **p < 0.01. Values are presented as the mean ± standard error of the mean (SEM).

To determine whether Piezo1 is involved in the development of AF, the expression levels of Piezo1 in LAAs of AF or SR patients were measured (for patient characteristics, see Table 1). As shown in Figures 1A,B, the protein levels of Piezo1, CaM, and Src were significantly greater in the LAAs of AF patients than the SR controls (0.46 ± 0.10 vs. 1.32 ± 0.11, *p* < 0.01; 0.60 ± 0.12 vs. 1.13 ± 0.12, *p* < 0.01; 0.63 ± 0.12 vs. 1.11 ± 0.09, *p* < 0.01; for Piezo1, CaM and Src, respectively), while Cav1.2 protein levels were lower in the LAAs of AF patients than the SR controls (0.97 ± 0.08 vs. 0.57 ± 0.10, *p* < 0.01; Figure 1A). These results indicate that Piezo1, CaM, and Src might participate in the decrease of atrial I_Ca,L_ in AF.

**Table 1:** Baseline characteristics of patients.

	**SR**	**AF**
*n*	10	10
Men (*n*)	6	5
Age (y)	50.60 ± 3.94	47.8 ± 4.59
SBP(mmHg)	112.5 ± 5.77	121.3 ± 7.73
DBP(mmHg)	66.5 ± 3.27	78.5 ± 4.20*
LAD (mm)	42.8 ± 2.70	51.10 ± 2.75*
EF (%)	46.7 ± 7.13	58.00 ± 5.28
AVR (*n*)	1	1
MVR (*n*)	3	7
β-blocker (*n*)	4	5
Digitalis (*n*)	5	8
Diuretics (*n*)	5	7

*p < 0.05 vs. SR. Values are mean ± SEM. n, number of patients; SBP, systolic blood pressure; DBP, diastolic blood pressure; LAD, left atria diameter; EF, ejection fraction; AVR, aortic valve replacement; MVR, mitral valve replacement; SR, sinus rhythm; AF, atrial fibrillation.

### Effects of Hypertension on the Depression of I_Ca,L_ and Incidence of AF in Wistar Rats and SHRs

#### Electrophysiological Characteristics and Incidence of AF in Wistar Rats and SHRs

**Figure 2:**
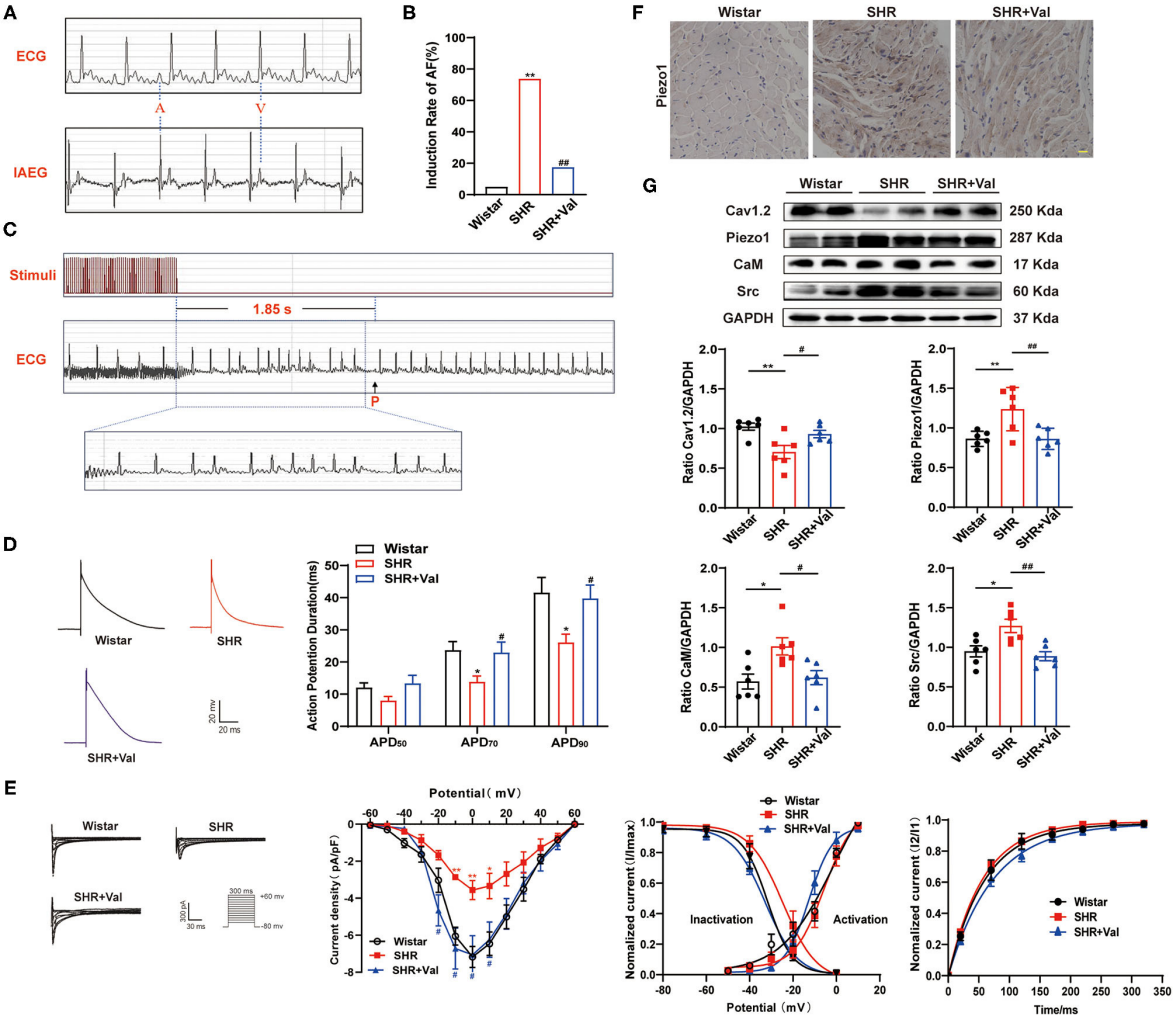
Effects of hypertension on the incidence of AF, I_Ca,L_, and Piezo1 expression in Wistar rats and SHRs with and without Val treatment. **(A)** Representative baseline surface ECG and intra-atrial electrocardiogram (IAEG); **(B)** The incidence AF in Wistar rats and SHRs treated with and without Val treatment (*n* = 8). **p < 0.01 vs. Wistar rat; ^*##*^ *p* < < 0.01 vs. SHRs. **(C)** Typical surface ECG recordings of rats with AF that spontaneously reverted to SR and typical disorganized amplification of atrial waves (f wave). **(D)** Representative traces of AP in atrial myocytes from Wistar rats, SHRs, and SHR + Val groups and a histogram of APD in atrial myocytes from each group (*n* = 12–15 myocytes from 3–4 rats). *p < 0.05 vs. Wistar rat; ^#^*p* < 0.05 vs. SHRs. **(E)** Representative traces of I_Ca,L_ (pulse protocol, inset), corresponding current-voltage relationship, mean data for voltage dependence activation, inactivation, and time course of recovery current for I_Ca,L_ in atrial myocytes of each group (*n* = 8–14 myocytes from 3–4 rats). **(F)** Representative examples of immunohistochemical analysis of LA tissues from Wistar rats and SHRs treated with and without Val using Ab against Piezo1. Scale bar, 20 μm. **(G)** Representative western blots and densitometric analysis of Cav1.2, Piezo1, CaM, and Src in LA tissues of Wistar rats and SHRs. GAPDH was the internal control. Values are presented as the mean ± SEM.

Wistar rats and SHRs were used to investigate the association of hypertension with the development of AF. Electrophysiological analysis showed that BP of SHRs was significantly higher than that of the control group, which was reversed by Val (Table 2). After rapid atrial pacing, the incidence of AF was significantly increased in SHRs as compared to Wistar rats (73.75 vs. 5.00%, respectively). Notably, administration of Val decreased the incidence of AF by 17.50% (Figures 2A–C). These findings indicate that hypertension plays an essential role in the risk of AF.

**Table 2:** General characteristics and electrophysiological analysis of rats.

	**Wistar**	**SHR**	**SHR + Val**
*n*	8	8	8
SBP (mmHg)	132.8 ± 1.70	189.8 ± 2.22**	162.9 ± 2.60^##^
DBP (mmHg)	102.8 ± 3.33	140.0 ± 3.21**	117.9 ± 5.01^##^
MAP (mmHg)	114.1 ± 2.64	156.2 ± 2.51**	132.7 ± 3.97^##^
HR (bpm)	404.6 ± 21.33	402.0 ± 21.11	379.4 ± 21.52
PWD (ms)	21.08 ± 0.64	26.5 ± 2.58*	23.06 ± 0.68
PR interval (ms)	41.91 ± 2.23	46.56 ± 1.88	43.13 ± 1.54
Incidence of AF (%)	5.00%	73.75%**	17.50%^##^
Mean AF duration (s)	0.44 ± 0.22	2.40 ± 0.48*	1.75 ± 0.61

SBP, systolic blood pressure; DBP, diastolic blood pressure; MAP, mean arterial pressure; HR, heart rate; PWD, P wave duration; AF, atrial fibrillation. *p < 0.05, **p < 0.01 vs. Wistar rats, ^##^p < 0.01 vs. SHRs. Values are mean ± SEM.

#### I_Ca,L_ of Atrial Myocytes and Expression of Piezo1 and Pathway-Related Proteins in the LA Tissues of Wistar Rats and SHRs

The APD of atrial myocytes at 50, 70, and 90% repolarization (APD_50_, APD_70_, and APD_90_, respectively) was recorded (Figure 2D). As compared to the control group of Wistar rats, APD_70_ and APD_90_ were shorter in SHRs (23.69 ± 2.66 vs. 13.84 ± 1.82 ms, *p* < 0.05; 41.54 ± 4.72 vs. 26.08 ± 2.58 ms, *p* < 0.05, respectively; Figure 2D), which was ameliorated by the Val (13.84 ± 1.82 vs. 22.95 ± 3.25 ms, *p* < 0.05; 26.08 ± 2.58 vs. 39.79 ± 4.14 ms, *p* < 0.05, respectively; Figure 2D). Meanwhile, the peak amplitude of I_Ca,L_ was significantly decreased in SHRs as compared to Wistar rats (-3.55 ± 0.51 vs. −7.16 ± 0.57 pA/pF, respectively, *p* < 0.01). This effect was also ameliorated by Val in SHRs (−7.05 ± 1.03 pA/pF, *p* < 0.05; Figure 2E). There were no significant differences in I_Ca,L_ activation, inactivation, and recovery among the three groups.

Protein expression of Cav1.2, which changed paralleled with the corresponding current, was decreased in SHRs and ameliorated by Val treatment (0.70 ± 0.08 in Wistar rat group vs. 1.02 ± 0.04 in SHRs, *p* < 0.01; 0.93 ± 0.04 in SHR+Val group, *p* < 0.05; Figure 2G). Meanwhile, the protein levels of Piezo1, CaM, and Src were increased in SHRs as compared to Wistar rats, and reversed by the Val treatment (Figure 2G). Immunohistochemistry indicated the same result of Piezo1 in the LA tissues of rats (Figure 2F).

These results are consistent with prior studies that long-term high-pressure is involved in ion channel remodeling and is affected the APD to increase AF susceptibility, which was relieved by Val. Such demonstration might be associated with activation of Piezo1, CaM, and Src.

### Effects of HHP on I_Ca,L_ and the Expression of Piezo1 and Pathway-Related Proteins in HL-1 Cells

**Figure 3:**
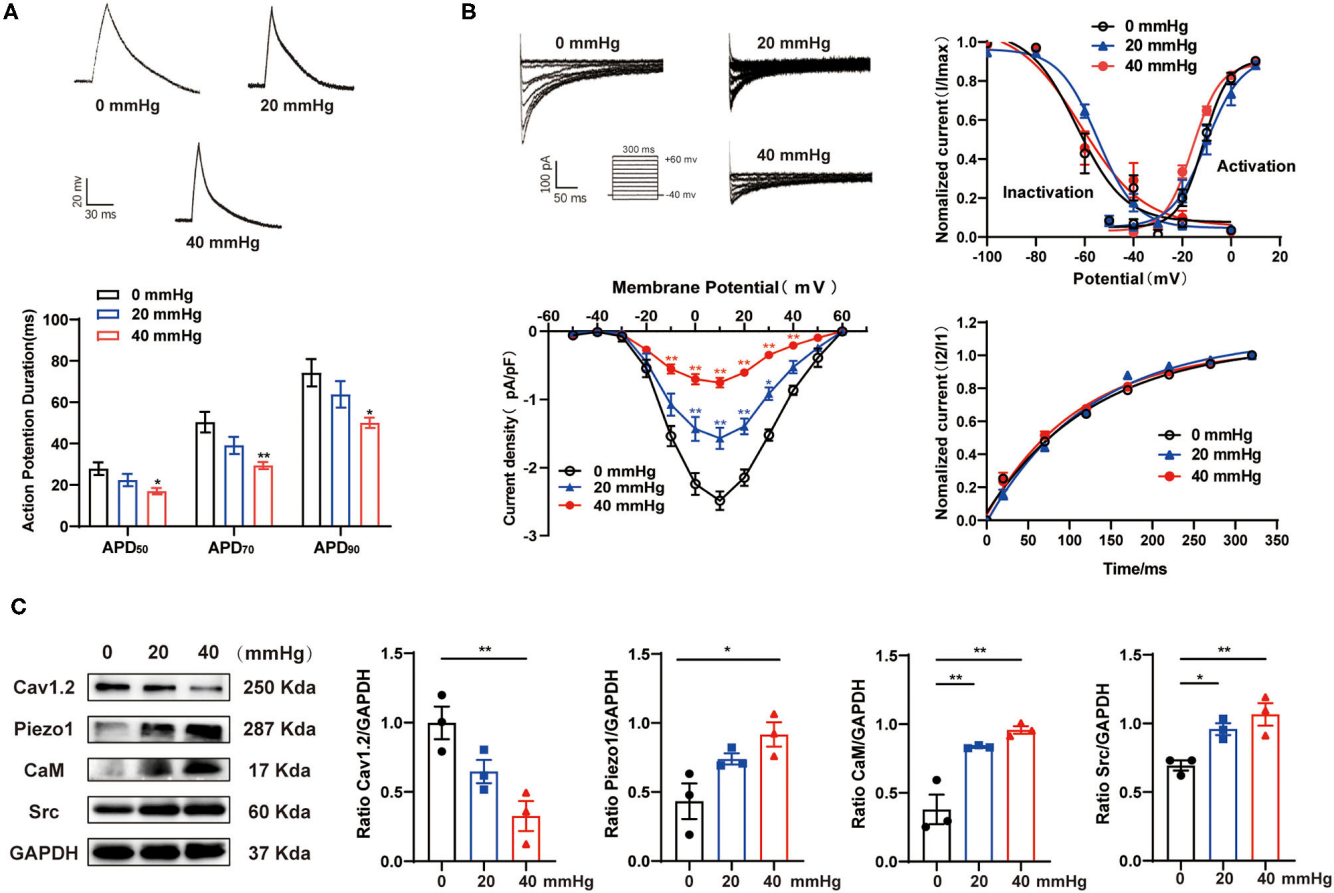
Effect of HHP on the depression of I_Ca,L_ in HL-1 cells. **(A)** Representative traces of AP in HL-1 cells under various hydrostatic pressures (0, 20, and 40 mmHg) for 24 h. APD_50_, APD_70_, and APD_90_ of HL-1 cells were calculated (*n* = 9, 10, and 7 at 0, 20, and 40 mmHg, respectively). *p < 0.05, **p < 0.01 vs. 0 mmHg. **(B)** Representative traces (pulse protocol, inset), corresponding current-voltage relationship, mean data for voltage dependence activation, inactivation, and time course of recovery current for I_Ca,L_ (*n* = 8–18 at 0, 20, and 40 mmHg, respectively). *p < 0.05, **p < 0.01 vs. 0 mmHg. **(C)** Representative western blots and densitometric analysis of Cav1.2, Piezo1, CaM, and Src in HL-1 cells under various hydrostatic pressure (0, 20, and 40 mmHg) for 24 h. GAPDH was used as an internal control. Values are presented as the mean ± SEM.

To further investigate the influence of hypertension on the decrease of I_Ca,L_ and related molecular signaling pathways, atrium-derived HL-1 cells were cultured under HHP conditions. As shown in Figure 3A, HHP (40 mmHg) significantly decreased the APD_50_, APD_70_, and APD_90_ values as compared to the control cells (0 mmHg) (27.88 ± 3.09 vs. 16.97 ± 1.53 ms, *p* < 0.05; 50.39 ± 5.01 vs. 29.42 ± 1.74 ms, *p* < 0.01; 74.24 ± 6.61 vs. 50.04 ± 2.50 ms, *p* < 0.05, respectively). Correspondingly, the peak amplitudes of I_Ca,L_ at 10 mV had decreased at hydrostatic pressures of 20 and 40 mmHg (−2.48 ± 0.14 pA/pF at 0 mmHg vs. −1.57 ± 0.16 pA/pF at 20 mmHg vs. −0.75 ± 0.07 pA/pF in 40 mmHg, *p* < 0.01, n = 9–11; Figure 3B), while there was no significant difference in activation, inactivation, and recovery of I_Ca,L_ among three groups (Figure 3B).

As compared to 0 mmHg, Cav1.2 protein expression was significantly decreased in HL-1 cells at 40 mmHg (1.00 ± 0.12 vs. 0.33 ± 0.11, respectively; *p* < 0.01), while the expression levels of Piezo1, CaM, and Src were significantly increased (0.43 ± 0.13 vs. 0.92 ± 0.09, *p* < 0.05; 0.38 ± 0.11 vs. 0.96 ± 0.03, *p* < 0.01; 0.69 ± 0.04 vs. 1.07 ± 0.08, *p* < 0.01, respectively; Figure 3C). These results indicate the crucial role of hydrostatic pressure in the decrease of I_Ca,L_, shortening of the APD, and activation of the Piezo1/CaM/Src signaling pathway after HHP stimulation.

### Piezo1 Is Involved in the Decrease of I_Ca,L_ of HL-1 Cells Induced by HHP

**Figure 4:**
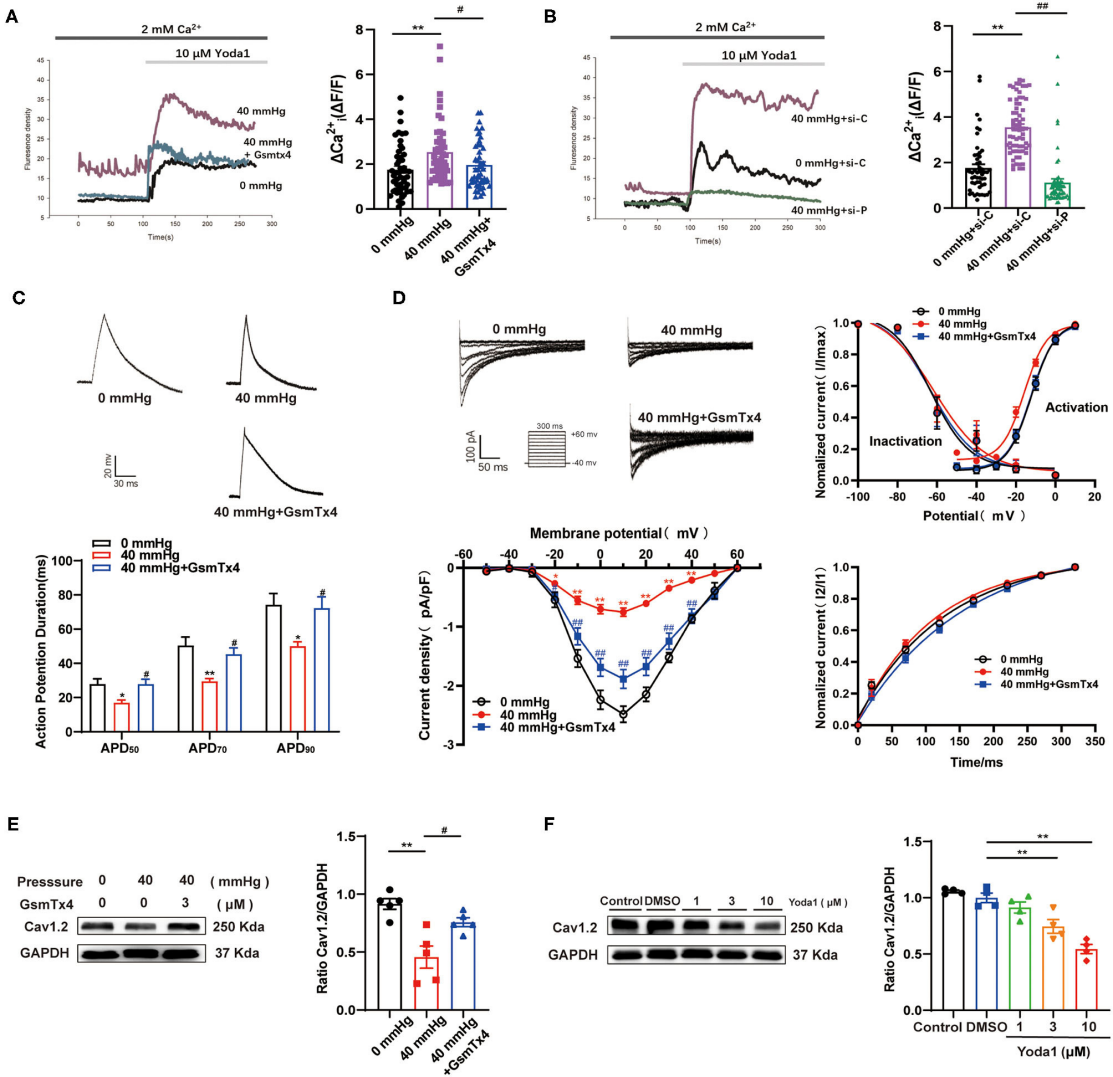
The effects of Piezo1 on perceiving HHP and mediating the decrease of I_Ca,L_. **(A,B)** Representative Ca^2+^ traces and Δ${\text{Ca}}_{\text{i}}^{2+}$(ΔF/F) are shown. Ca^2+^ entry was evoked by 10 μm Yoda1 in HL-1 cells stimulated by HHP in the presence or absence of the Piezo1 inhibitor GsmTx4 (*n* = 50) or siRNA specifically knockdown Piezo1 (*n* = 53–58). si-C, scrambled (control) siRNA; si-P, siRNA directed against Piezo1. **(C)** Representative traces of AP in HL-1 cells stimulated by 40 mmHg pressure with and without GsmTx4 treatment (3.0 μM) and APD_50_, APD_70_, and APD_90_ of HL-1 cells were calculated (*n* = 9, 7, and 11 at 0, 40, and 40 mmHg + GsmTx4). *p < 0.05, **p < 0.01 vs. 0 mmHg; ^#^*p* < 0.05 vs. 40 mmHg. **(D)** Representative traces (pulse protocol, inset), corresponding current–voltage relationship, mean data for voltage dependence activation, inactivation, and time course of recovery current for I_Ca,L_ in HL-1 cells stimulated by 40 mmHg pressure with and without GsmTx4 treatment (*n* = 9–15). **p < 0.01 vs. 0 mmHg; ^##^*p* < 0.01 vs. 40 mmHg. **(E)** Representative blots and densitometry analysis of Cav1.2 in HL-1 cells stimulated by 40 mmHg pressure with and without GsmTx4 treatment. **(F)** Representative blots and densitometry analysis of Cav1.2 in Yoda1 stimulation at different dosages (1, 3, and 10 μM) for 48 h. GAPDH was used as an internal control. Values are presented as the mean ± SEM.

To further confirm the role of Piezo1 in atrial mechanotransduction, the effects of GsMTx4, an inhibitor that explicitly targets cation MSCs, and small interfering RNA (siRNA) against Piezo1 on Yoda1-induced Ca^2+^ entry were investigated in HL-1 cells treated with HHP. Yoda1 (10 μM) increased peak [Ca^2+^]_i_ and caused sustained [Ca^2+^]_i_ elevations in HL-1 cells, which was more pronounced in cells treated the 40 mmHg high-pressure groups (*p* < 0.01) (fluorescence intensity calculated at 6 min after Yoda1 application) (Figures 4A,B). Inhibition or knockdown of Piezo1 strongly inhibited HHP-induced increases in [Ca^2+^]_i_ (*p* < 0.05; Figures 4A,B), which provided evidence that Piezo1 was activated by HHP.

APD shortening and decreased I_Ca,L_ induced by HHP were ameliorated by blocking Piezo1 channels with GsMTx4 (Figures 4C,D). No significant difference was observed in I_Ca,L_ activation, inactivation, and recovery among the three groups (Figure 4D). Consistently, Cav1.2 protein expression was reversed in HL-1 cells stimulated by HHP in the presence of GsMTx4 (0.46 ± 0.10 vs. 0.76 ± 0. 04, *p* < 0.05; Figure 4E). As compared to the control group treated with DMSO, Cav1.2 expression was downregulated by different concentrations (3 and 10 μM) of Yoda1 (1.00 ± 0.04 vs. 0.75 ± 0.06 vs. 0.54 ± 0.04, respectively; *p* < 0.01; Figure 4F). These results provided strong evidence that Piezo1 was involved in the decrease of I_Ca,L_ in response to HHP.

### Piezo1 Participated in the Depression of I_Ca,L_ Induced by HHP via. CaM/Src

#### CaM and Src Are Downstream Signaling Molecules of Piezo1

**Figure 5:**
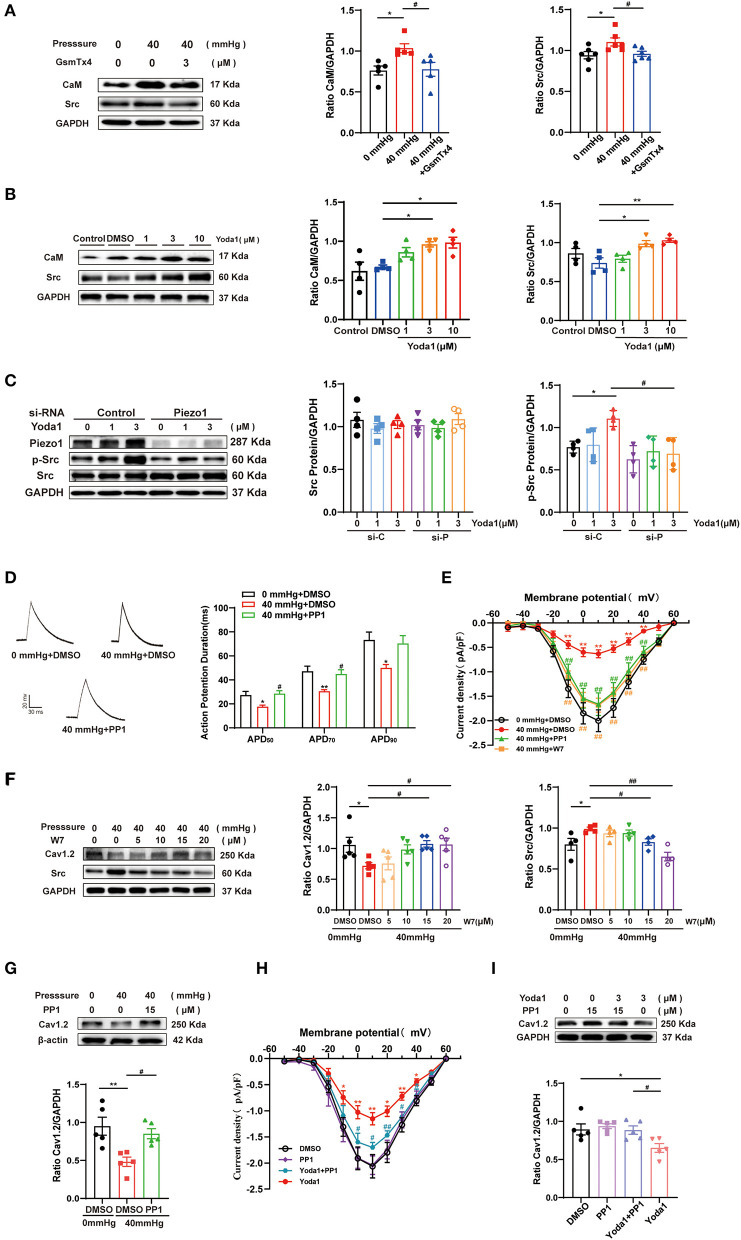
Effect of CaM/Src on the decrease of I_Ca,L_ induced by HHP or Yoda1 stimulation. **(A)** Representative blots and densitometry analysis of CaM and Src in HL-1 cells stimulated by 40 mmHg pressure with and without GsmTx4 treatment. **(B)** Representative blots and densitometry analysis of CaM and Src in Yoda1 stimulation at different dosages (1, 3, and 10 μM) for 48 h. **(C)** Representative blots and densitometry analysis of Src and p-Src (*n* = 4) in HL-1 cells transfected with scrambled (control) siRNA or siRNA directed against Piezo1 for 48 h, then treated with Yoda1 at different dosages (0, 1, and 3 μM) for 15 min. **(D)** Representative traces of AP and histogram of APD in HL-1 cells (*n* = 7–8). *p < 0.05 vs. 0 mmHg + DMSO. ^#^*p* < 0.05 vs. 40 mmHg + DMSO. **(E)** Current–voltage relationship for I_Ca,L_ (*n* = 9–17) in HL-1 cells stimulated by 40 mmHg pressure treated with 15 μM PP1 or W7. *p < 0.05 vs. 0 mmHg + DMSO. ^#^*p* < 0.05 vs. 40 mmHg + DMSO. **(F)** Representative blots and densitometry analysis of Cav1.2 and Src in HL-1 cells stimulated by 40 mmHg pressure treated with W7 under different concentrations (5, 10, 15, and 20 μM). **(G)** Representative blots and densitometry analysis of Cav1.2 in HL-1 cells stimulated by 40 mmHg pressure treated with PP1 (15 μM). **(H)** Current-voltage relationship for I_Ca,L_ (*n* = 8–10) in HL-1 cells stimulated by Yoda1(3 μM) treated with PP1. *p < 0.05, **p < 0.01 vs. DMSO. ^#^*p* < 0.05, ^##^*p* < 0.01 vs. Yoda1. **(I)** Representative blots and densitometry analysis of Cav1.2 in Yoda1(3μM) -stimulated HL-1 cells treated with PP1 (15 μM). GAPDH was used as an internal control. Values are presented as the mean ± SEM.

GsMTx4 and Yoda1 were used to determine the relationship between CaM/Src and Piezo1 and to identify the signaling pathways underlying HHP-induced AF. As expected, the protein expression level of CaM and Src were downregulated in HL-1 cells stimulated by HHP in the presence of GsMTx4 (1.04 ± 0.05 vs. 0.78 ± 0.08, *p* < 0.05; 1.10 ± 0.05 vs. 0.96 ± 0.03, *p* < 0.05; for CaM and Src; Figure 5A). As compared to the DMSO-treated control group, Yoda1 stimulated an increase in the expression levels of CaM and Src in a concentration-dependent manner at 3 and 10 μM (0.67 ± 0.02 vs. 0.96 ± 0.03 *p* < 0.05, vs. 0.98 ± 0.07, *p* < 0.05; 0.74 ± 0.07 vs. 0.99 ± 0.04, *p* < 0.05; and 1.03 ± 0.03, *p* < 0.01, respectively; Figure 5B). These results indicate that CaM and Src are downstream signaling molecules of Piezo1 and might be involved in the decrease of I_Ca,L_. To further investigate the specificity of the signaling pathways involved in I_Ca,L_ downregulation induced by Piezo1, p-Src levels were measured in HL-1 cells treated with 0, 1, or 3 μM of Yoda1 for 10–15 min with and without Piezo1 siRNA. As shown in Figure 5C, p-Src expression was significantly increased by stimulation with 3 μM Yoda1, while knockdown of Piezo1 abolished this effect. Thus, activation of Piezo1 by chemical activation (Yoda1) is an essential process for downstream signaling of CaM/Src and activation is coupled to Src phosphorylation.

#### Effects of CaM/Src on HHP/Piezo1 Activation-Induced the Decrease of I_Ca,L_

The CaM antagonist N-(6-aminohexyl)-5-chloro-1-naphthalenesulfonamide(W-7) or Src kinase-specific inhibitor PP1 was used to confirm the roles of CaM and Src in the decrease of I_Ca,L_. The shortening of APD_50_, APD_70_, and APD_90_ and the depression of I_Ca,L_ peak amplitudes induced by HHP stimulation was alleviated by 15 μM PP1 or W7 treatment (−2.00 ± 0.22 pA/pF for 0 mmHg + DMSO vs. −0.63 ± 0.08 pA/pF for 40 mmHg + DMSO vs. −1.66 ± 0.23 pA/pF for 40 mmHg + PP1 and −1.68 ± 0.15 pA/pF for 40 mmHg + W7 at 10 mV, *n* = 9–15, *p* < 0.01; Figures 5D,E). However, the kinetic properties of I_Ca,L_ were not modified (Supplementary Figure 1).

Consistent with the current results, both W7 and PP1 improved the decrease in Cav1.2 expression induced by HHP (0.72 ± 0.05 vs. 1.08 ± 0.05 at 15 μM and 1.07 ± 0.10 at 20 μM, *p* < 0.05; 0.48 ± 0.06 vs. 0.85 ± 0.07, *p* < 0.05, respectively; Figures 5F,G). W7 alleviated HHP-induced upregulation of Src expression (0.99 ± 0.02 vs. 0.83 ± 0.04 at 15 μM and 0.65 ± 0.05 at 20 μM, *p* < 0.05 and *p* < 0.01; Figure 5F). This result strongly indicates that CaM is upstream of Src.

The role of the Piezo1 agonist Yoda1 is similar to HHP stimulation. A decrease in peak amplitudes of I_Ca,L_ during Yoda1 stimulation was also alleviated with 15 μM PP1 (Figure 5H), while no significant difference was observed in I_Ca,L_ channel characteristics among the three groups (Supplementary Figure 1). Similarly, PP1 was found to upregulate the decrease of Cav1.2 under Yoda1 stimulation (Figure 5I). These data established that CaM and Src play crucial roles in HHP- and Piezo1-induced I_Ca,L_ depression.

### HHP/Piezo1-Induced Pitx2 Activation Is the Consequence of Src Activation

**Figure 6:**
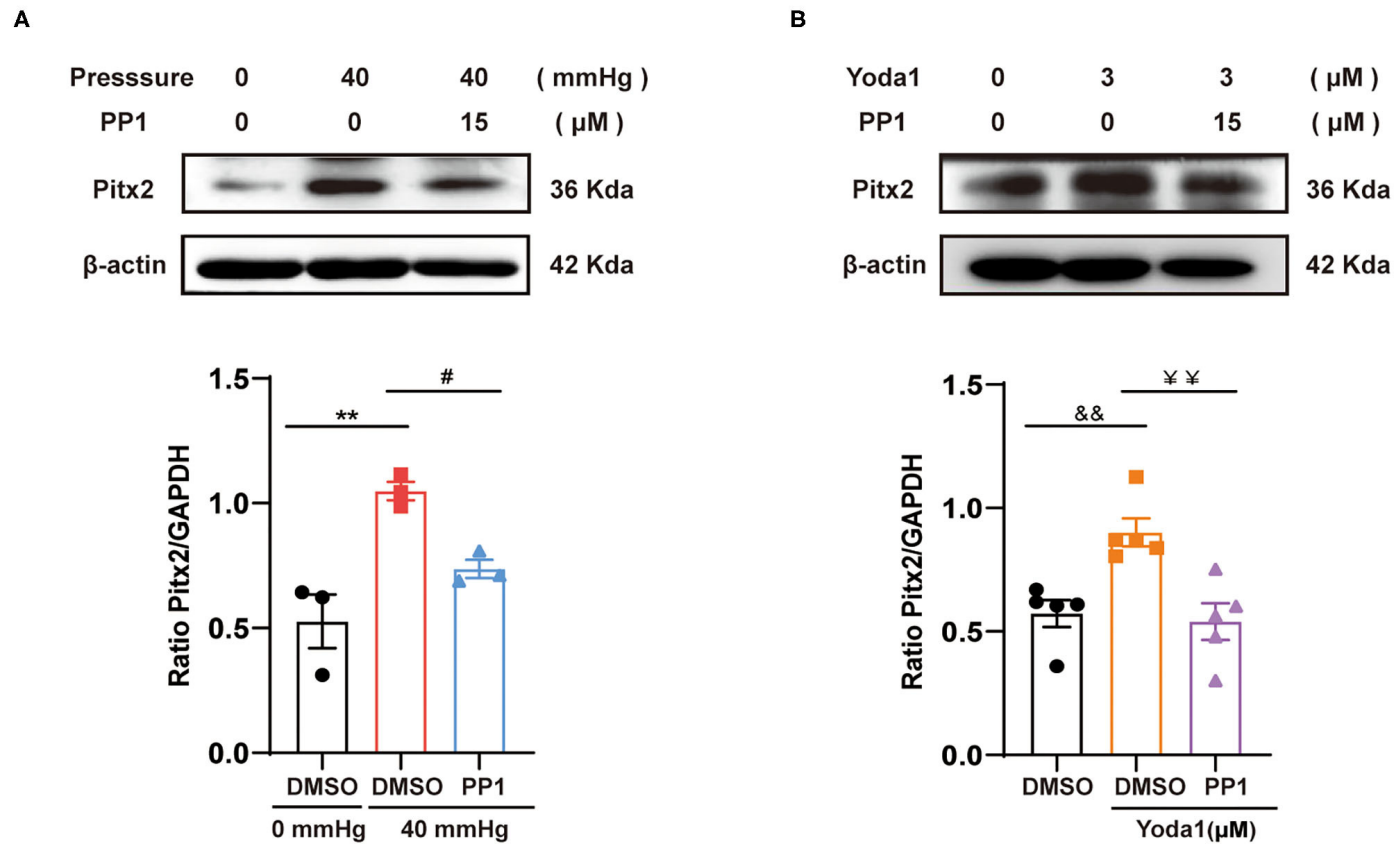
Effect of Src on the expression of Pitx2 induced by HHP or Yoda1 stimulation. Representative blots and densitometry analysis of Pitx2 in HL-1 cells stimulated by HHP **(A)** or Yoda1 **(B)** treated with and without PP1 (15 μM). β-actin was used as an internal control. **p < 0.01, ^#^*p* < 0.05; ^&&^*p* < 0.01, ^¥¥^*p* < 0.01. Values are presented as the mean ± SEM.

As mentioned above, Src was involved in the depression of I_Ca,L_ and downregulation of Cav1.2; however, the specific mechanisms remain unclear. Pitx2, a transcription factor, has been found to be elevated in AF patients. Recent evidence suggests that Pitx2 plays a role in the pathophysiology of AF and is closely related to the increase in I_Ks_ as well as the decrease in I_Ca,L_. So, in the present study, the relationship between Src and Pitx2 in response to HHP/Piezo1 stimulation was explored. The results showed that Pitx2 expression was increased in the context of Piezo1 activation induced by HHP and Yoda1 (0.53 ± 0.11 vs. 1.05 ± 0.04, *p* < 0.01; 0. 57 ± 0.05 vs. 0.90 ± 0.06, *p* < 0.01, respectively, Figure 6), while this trend was alleviated by PP1 treatment (1.05 ± 0.04 vs. 0.74 ± 0.04, *p* < 0.05; 0.90 ± 0.06 vs. 0.54 ± 0.07, *p* < 0.01, respectively, Figure 6), indicating that Pitx2 operates downstream of Src activation induced by HHP and Piezo1.

## Discussion

**Figure 7:**
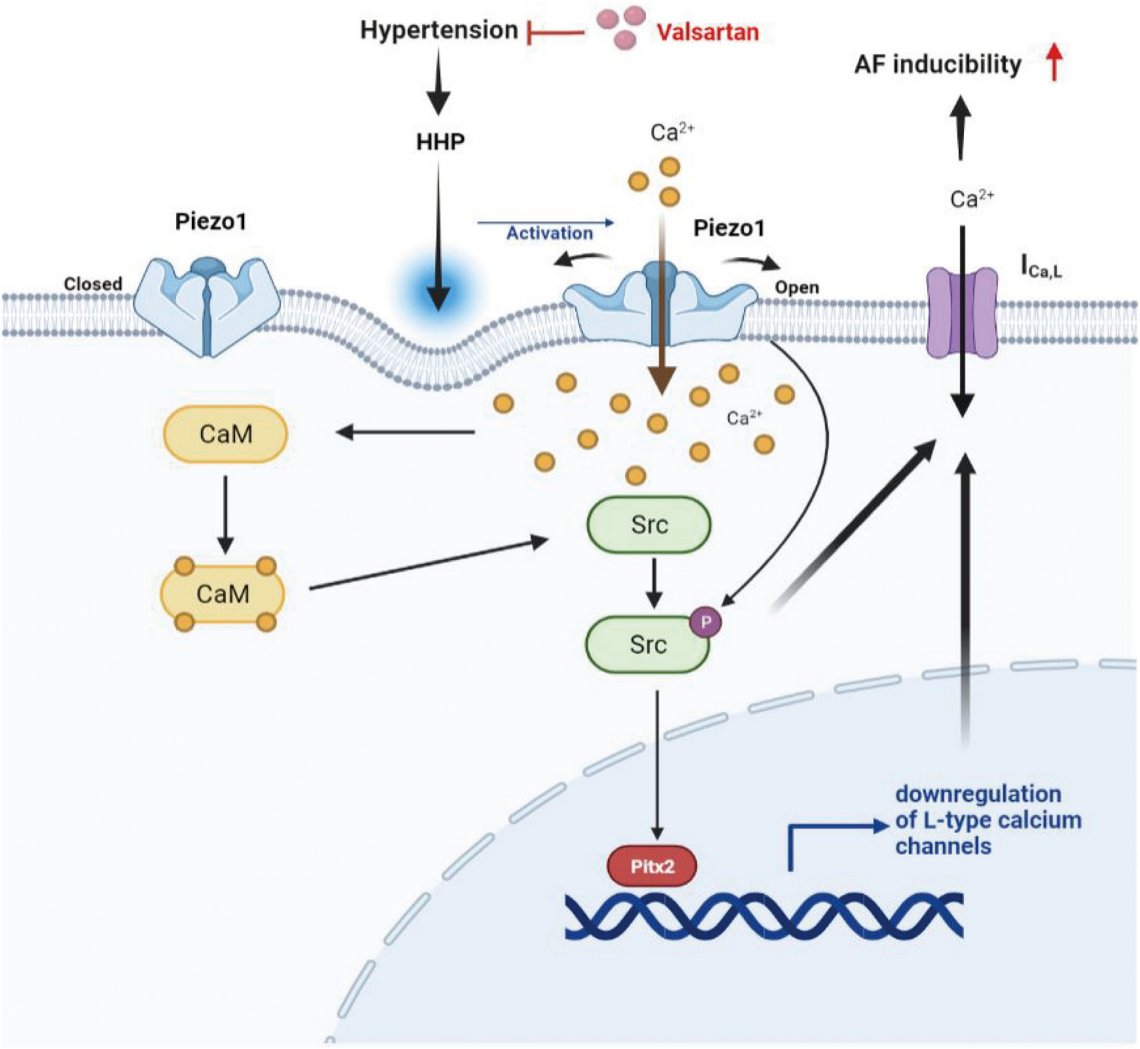
Schematic representation of the mechanism for the decrease of I_Ca,L_ induced by HHP. Piezo1 activated by HHP depressed I_Ca,L_ contributing to increased AF susceptibility through the CaM/Src/Pitx2 pathway.

The significant findings of this study are that human and rat atrial tissues express Piezo1 channels and that are activated by hypertension and that in atrial myocytes, HHP-induced activation of Piezo1 was coupled to the CaM/Src/Pitx2 pathway and participated the decrease of I_Ca,L_ contributing to APD shortening and an increase in susceptibility to AF. These data establish a link between atrial I_Ca,L_ depression in AF and Piezo1 through activation of the its downstream molecular signals CaM, Src, and Pitx2 after HHP-induced stimulation. The schematic representation of these mechanisms was shown in Figure 7.

AF is self-maintained and is progressive in nature (32). The maintenance of AF has been associated with the interaction between electrical and structural remodeling and the independent effects of both factors (33). I_Ca,L_ decreasing and APD shortening are critical components of electrical remodeling. Our previous study has demonstrated that I_Ca,L_ was depressed (34) and APD was shortened in atrial myocytes of patients with AF as compared with those of SR controls (35). In the present study, Cav1.2 protein expression was parallel with the current results in AF patients. Hypertension is a significant risks of AF. In the present study, AF inducibility of SHR increased significantly accompanied by decreased I_Ca,L_ in atrial myocytes and Cav1.2 protein expression in atrial tissue and APD shortening, which could be reversed by val treatment. However, how hypertension, especially HHP, induces I_Ca,L_ decreasing and APD shortening is unclear.

Piezo1, a member of the newly discovered family of MSCs (36), has been shown to participate in the mechanosensation of various biological processes. However, it is unclear whether Piezo1 is involved in hypertension-induced AF. The results showed that Piezo1 protein expression was increased in the atria of both AF patients and SHRs, which was reversed by Val, an effective antihypertensive drug, indicating that Piezo1 might participate in hypertension-induced AF. However, due to limited human specimens, it was not possible to perform subgroup analysis of BP in SR and AF patients. The trend of BP in the baseline characteristics of patients was consistent with the expression of Piezo1. In addition, elevated LA pressure is a prominent feature of AF (37), demonstrating that Piezo1 in atrial myocytes could respond to long-term pressure loads and might play a crucial part in AF. Hypertension can lead to further increases in atrial pressure. In a state of hypertension, the main changes in mechanical stress are increased cyclic stretch and hydrostatic pressure. A recent study reported that Piezo1 channels in HL-1 atrial myocytes were activated by stretching stimulation *in vitro* (22). However, whether hydrostatic pressure can activate Piezo1 in atrial myocytes remains unclear. Related studies have suggested out that Piezo1 acts as a receptor for hydrostatic pressure in mesenchymal stem cells, goblet cells, and stem cells from human exfoliated deciduous teeth (38–40). Elevated microvessel hydrostatic pressure in the lung results in the opening of Piezo1, which mediates disruption of endothelial barrier, leading to pulmonary edema (41). The result of the present study further found that Piezo1 in atrial myocytes can respond to hydrostatic pressure and affect the decrease of I_Ca,L_. Piezo1 expression was increased with enhanced Piezo1 channel function, as determined by Ca^2+^ entering atrium-derived HL-1 cells in response to HHP, while inhibition or activation of the Piezo1 channels reversed and mimicked HHP-induced depression of I_Ca,L_. In addition, Piezo1 had no impact on the channel characteristics of I_Ca,L_, suggesting that Piezo1 depressed I_Ca,L_ by downregulating Cav1.2 expression. Collectively, these findings suggest that Piezo1 robustly promotes the depression of I_Ca,L_ in atrial myocytes in response to HHP.

The potential signaling pathways underlying Piezo1-stimulated I_Ca,L_ depression in atrial tissues were also explored. CaM, as a ubiquitously expressed and highly versatile Ca^2+^ sensor, regulates the function of many ion channels and enzymes (42). As illustrated by the results of the present study, CaM acts downstream of Piezo1, as CaM expression can be inhibited by GsmTx4 in response to HHP and increased by Yoda1, suggesting that the influx of Ca^2+^ through Piezo1 can activate CaM. In addition to promoting inactivation of the Cav1.2 complex (43, 44), inhibition of CaM reversed the decrease of I_Ca,L_ and downregulation of Cav1.2 in response to HHP, suggesting that CaM also influenced the expression of L-type calcium channel. Meanwhile, a previous study found that Ca^2+^/CaM can bind to and enhance the tyrosine kinase activity of c-Src (45). The results of the present study found that inhibition of CaM can decrease the expression of Src in response to HHP. Src, a member of Src-nPTKs family, has been implicated in AF (46–48), as Src inhibits single I_Ca,L_ in atrial myocytes by phosphorylation of critical tyrosine residues of Cav1.2 (30), which acts to regulate phosphorylation-dependent channels. Moreover, our previous study found that Src participates in decreasing I_Ca,L_ of atrial myocytes in response to HHP by regulating the expression of channel proteins (31). The results of the present study found that Src was downstream of Piezo1 and is phosphorylated by Piezo1, which regulates Cav1.2 through various mechanisms in response to HHP.

Although some studies have found that Src depresses I_Ca,L_ and decreases the expression of Cav1.2 in atrial myocytes, the underlying mechanism remain unclear. The gene-poor 4q25 region associated with AF (49–51) harbors the Pitx2 homeobox gene, which has been implicated in predisposition for AF (52, 53). Recent evidence suggests that Pitx2 mRNA expression was significantly higher in human atrial myocytes from AF patients than those with SR. Furthermore, the increased expression of Pitx2 decreased I_Ca,L_ and shortened the APD in atrial myocytes (54, 55). We further found that Pitx2 had elevated in response to HHP and Yoda1 stimulation, which was blocked by inhibiting Src, indicating that Pitx2 is downstream of Src and then participates in the decrease of I_Ca,L_ in the context of HHP and Piezo1 activation.

There were some potential limitations to this study that should be addressed. First, the number of samples was relatively small due to the difficulty of obtaining human specimens, which may have resulted in inherent bias. Second, the use of Piezo1 knockout mice would contribute to a better understanding of the role of Piezo1 in AF induced by hypertension. Finally, hydrostatic pressure devices produce continuous, rather than pulsating, high pressure in HL-1 cells.

In conclusion, this study is the first to establish Piezo1 as a functional mechanosensitive Ca^2+^-permeable ion channel in atrial myocytes that can be activated by HHP, leading to depression of I_Ca,L_. Specifically, CaM and Src acted downstream of Piezo1-mediated Ca^2+^ entry, resulting in increased Pitx2 expression, which is vital for the decrease of I_Ca,L_.

## Data Availability Statement

The original contributions presented in the study are included in the article/Supplementary Material, further inquiries can be directed to the corresponding author/s.

## Ethics Statement

The studies involving human participants were reviewed and approved by the Research Ethics Committee, Guangdong Provincial People's Hospital, Guangdong Academy of Medical Sciences (no. GDREC2017111H; Guangzhou, Guangdong, China). The patients/participants provided their written informed consent to participate in this study. The animal study was reviewed and approved by Research Ethics Committee of Sun Yat-sen University (Guangzhou, China), ethic code: SYSU-IACUC-2020-000220.

## Author Contributions

YF, FR, S-LW, and Y-MX designed the study. YF, QL, XL, G-HL, S-JK, and X-SL conducted the experiments and acquired the data. YF, FR, C-YD, Q-QL, HY, and YL performed data analysis. YF, QL, FR, and C-YD wrote and revised the manuscript. All authors contributed to the article and approved the submitted version.

## Funding

This work was financially supported by the High-level Hospital Construction Plan (Grant Nos. DFJH201808 and DFJH201925) and the National Natural Science Foundation of China (Grant Nos. 81670314 and 81870254).

## Conflict of Interest

The authors declare that the research was conducted in the absence of any commercial or financial relationships that could be construed as a potential conflict of interest.

## Publisher's Note

All claims expressed in this article are solely those of the authors and do not necessarily represent those of their affiliated organizations, or those of the publisher, the editors and the reviewers. Any product that may be evaluated in this article, or claim that may be made by its manufacturer, is not guaranteed or endorsed by the publisher.
