# Supplementary material for: Piezo1 Participated in Decreased L-Type Calcium Current Induced by High Hydrostatic Pressure via. CaM/Src/Pitx2 Activation in Atrial Myocytes
Source: Front Cardiovasc Med. 2022 Feb 17;9:842885. doi: 10.3389/fcvm.2022.842885 (PMC8891577; doi:10.3389/fcvm.2022.842885)
Supplement: Supplementary file 1 [file Data_Sheet_1.docx]

## Supplementary Figures

**
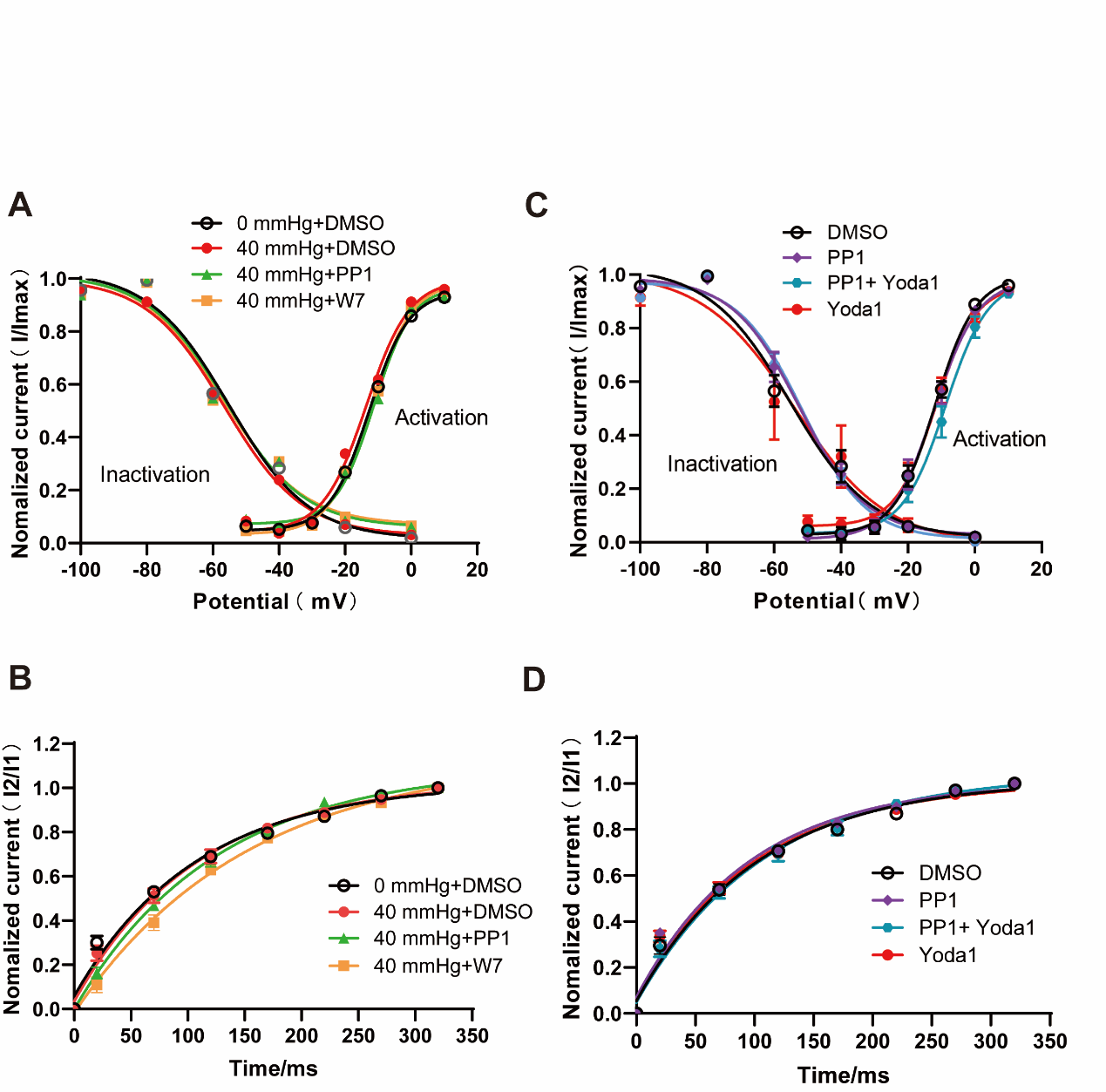
**

**Supplementary Figure 1.** Effect of CaM/Src on the channel characteristics of I_Ca,L_ induced by HHP stimulation or Yoda1 stimulation.

Mean data for voltage dependence activation, inactivation (A), and time course of recovery current (B) for I_Ca,L_ in 40 mmHg pressure-stimulated HL-1 cells treated with 15μM PP1 or W7 (n = 9–19). Mean data for voltage dependence activation, inactivation (C), and time course of recovery current (D) for I_Ca,L_ in Yoda1(3μM) stimulated HL-1 cells treated with PP1 (n = 5–16).
